# Classification of Different Degrees of Disability Following Intracerebral Hemorrhage: A Decision Tree Analysis from VISTA-ICH Collaboration

**DOI:** 10.3389/fneur.2017.00064

**Published:** 2017-02-28

**Authors:** Thanh G. Phan, Jian Chen, Richard Beare, Henry Ma, Benjamin Clissold, John Van Ly, Velandai Srikanth, the VISTA-ICH Collaboration, D. F. Hanley

**Affiliations:** ^1^Neurosciences, Monash Health, Melbourne, VIC, Australia; ^2^Department of Medicine, School of Clinical Sciences, Monash University, Clayton, VIC, Australia; ^3^Department of Medicine, Central Clinical School, Monash University, Frankston, VIC, Australia

**Keywords:** decision trees, intracerebral hemorrhage, outcome, disability evaluation, stroke

## Abstract

**Background and purpose:**

Prognostication following intracerebral hemorrhage (ICH) has focused on poor outcome at the expense of lumping together mild and moderate disability. We aimed to develop a novel approach at classifying a range of disability following ICH.

**Methods:**

The Virtual International Stroke Trial Archive collaboration database was searched for patients with ICH and known volume of ICH on baseline CT scans. Disability was partitioned into mild [modified Rankin Scale (mRS) at 90 days of 0–2], moderate (mRS = 3–4), and severe disabilities (mRS = 5–6). We used binary and trichotomy decision tree methodology. The data were randomly divided into training (2/3 of data) and validation (1/3 data) datasets. The area under the receiver operating characteristic curve (AUC) was used to calculate the accuracy of the decision tree model.

**Results:**

We identified 957 patients, age 65.9 ± 12.3 years, 63.7% males, and ICH volume 22.6 ± 22.1 ml. The binary tree showed that lower ICH volume (<13.7 ml), age (<66.5 years), serum glucose (<8.95 mmol/l), and systolic blood pressure (<170 mm Hg) discriminate between mild versus moderate-to-severe disabilities with AUC of 0.79 (95% CI 0.73–0.85). Large ICH volume (>27.9 ml), older age (>69.5 years), and low Glasgow Coma Scale (<15) classify severe disability with AUC of 0.80 (95% CI 0.75–0.86). The trichotomy tree showed that ICH volume, age, and serum glucose can separate mild, moderate, and severe disability groups with AUC 0.79 (95% CI 0.71–0.87).

**Conclusion:**

Both the binary and trichotomy methods provide equivalent discrimination of disability outcome after ICH. The trichotomy method can classify three categories at once, whereas this action was not possible with the binary method. The trichotomy method may be of use to clinicians and trialists for classifying a range of disability in ICH.

## Introduction

The incidence of intracerebral hemorrhage (ICH) is estimated at 24.6 per 100,000 per year (1). Despite the advances in stroke prevention and management, a recent meta-analysis has suggested that the incidence of ICH and its associated median 1 month mortality of 40% have not changed between 1980 and 2008 (1) (mortality is much lower in Japan). There have been many models (2–6) for prediction of poor outcome following ICH. These models emphasized the importance of the volume of the hematoma and the Glasgow Coma Scale (GCS) (5). Importantly, these models have focused mostly on predicting mortality or poor outcome (severe disability) (2, 4, 7–10). An earlier review in 2005 of prediction models for mortality showed that they have high specificity but low sensitivity and consequently do not perform well as a clinical triaging tool (2). A more recent systematic review a decade later revealed that tools based on ICH score had excellent ability to discriminate mortality with area under ROC curve between 0.8 and 0.87 (11). However, it is less certain if these tools can discriminate the outcome over the range of disabilities.

For prognostication, clinicians want to classify mild and severe disability outcomes. Because of the way the outcome data are dichotomized from the Rankin scale of disability, good outcome is buried in a much larger group comprising good and intermediate outcome (2, 3, 7). However, clinicians and trialists may also want to know about patients with mild, moderate, or severe disability (12). Surprisingly, few studies have focused on classification of mild disability (10). One may speculate that inclusion of patients who are likely to have mild disability in clinical trials may have been the reasons for the lack of positive results in these randomized control trials (13, 14).

Most models have been developed using regression-based methods such as logistic regression and only two studies (with small sample size) have used classification and regression tree (CART) analysis (15, 16). Regression-based methods are used for hypothesis testing and generation of predictive models. These models assume that all of the variables are required at once to formulate an accurate prediction.

**Figure 1:**
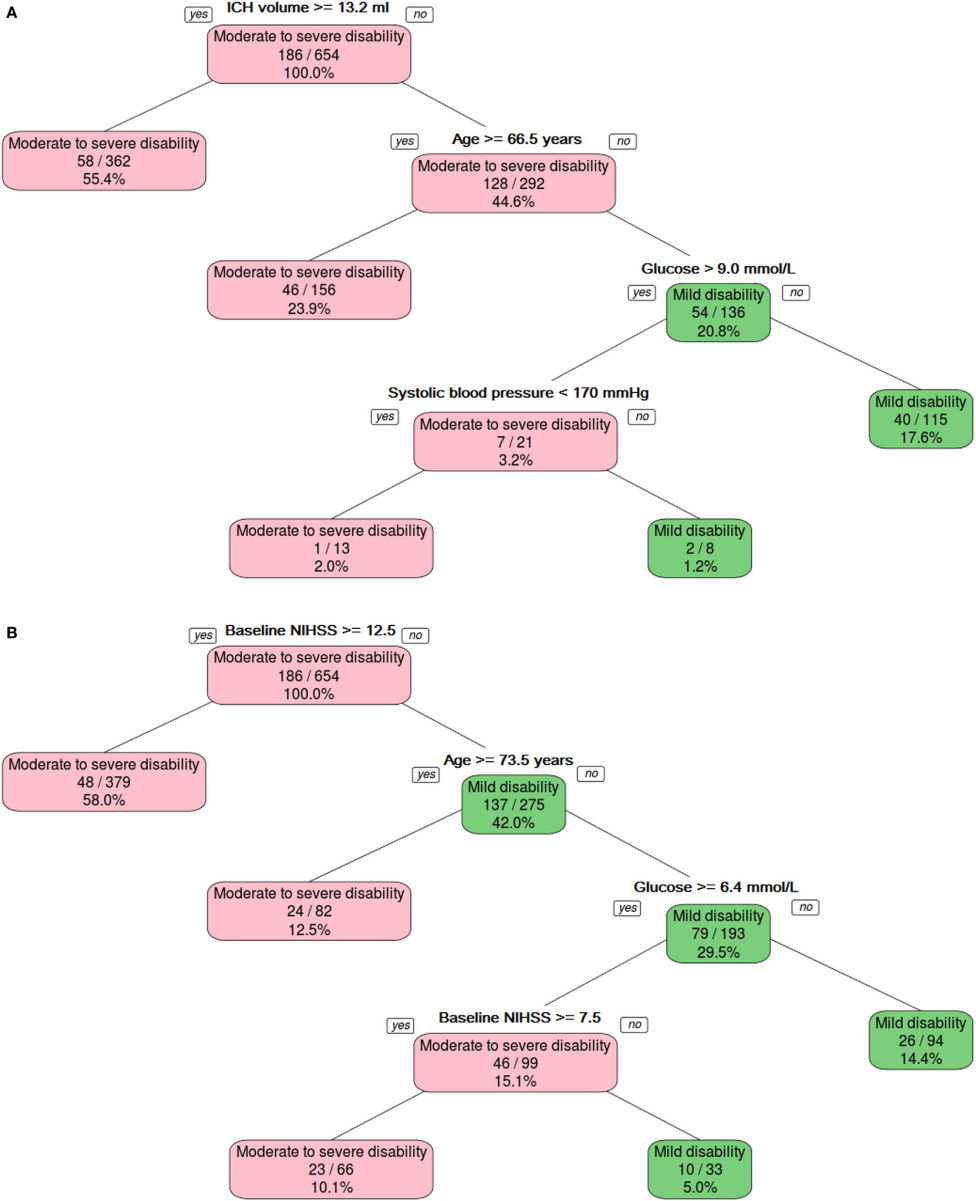
**The binary decision tree split the data into different categories on the basis of intracerebral hemorrhage (ICH) volume, age, and serum glucose level**. Panel **(A)** shows model developed without using NIHSS, and panel **(B)** shows model developed with NIHSS.

**Figure 2:**
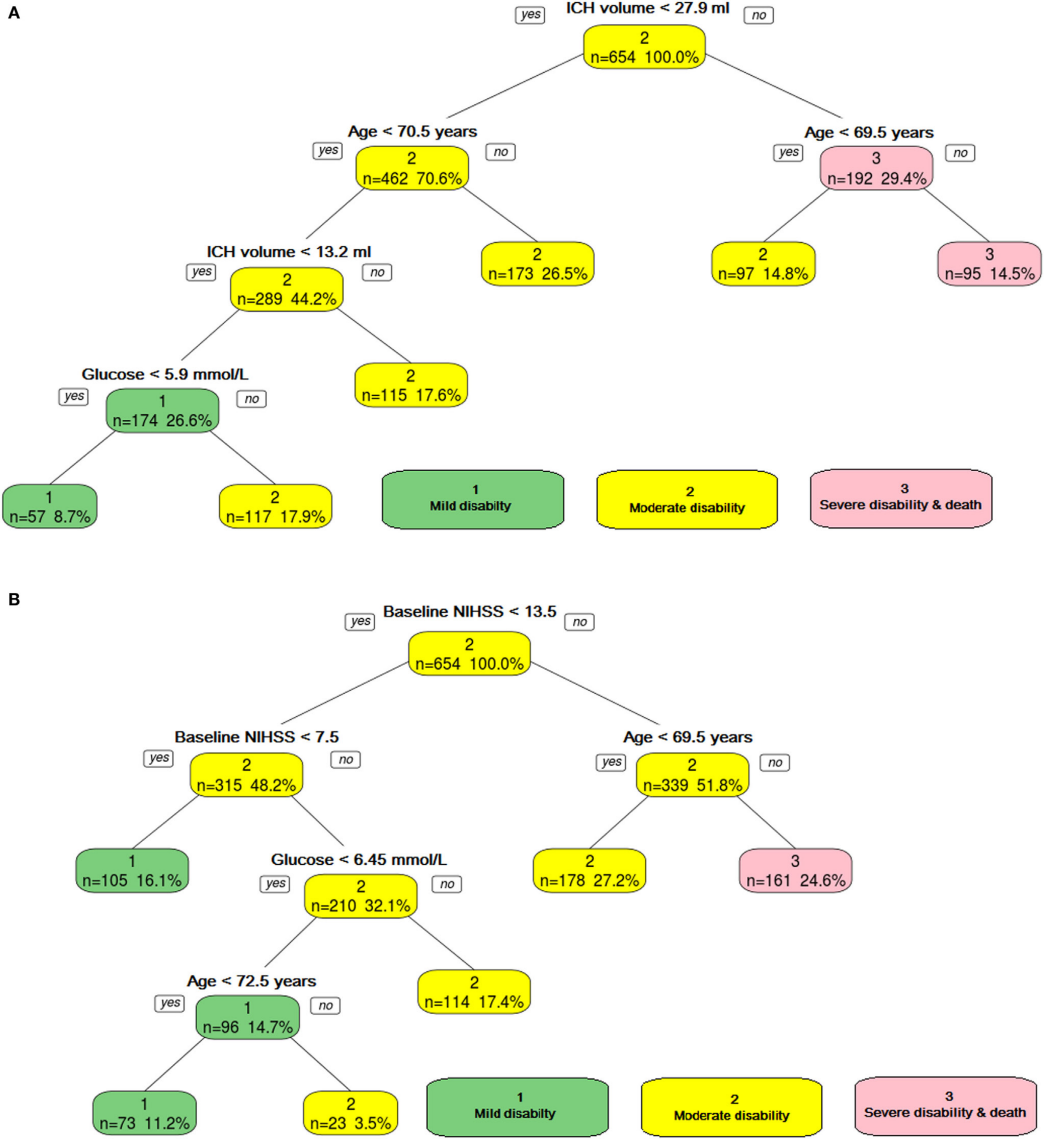
**The ordinal decision tree split the data into different categories on the basis of intracerebral hemorrhage (ICH) volume, age, and serum glucose level**. Panel **(A)** shows model developed without using NIHSS, and panel **(B)** shows model developed with NIHSS. Category 1—mild disability, Category 2—moderate, and Category 3—poor disability.

Such approaches assume that all of these beta coefficients are required at once to formulate a model. Due to the sequential nature of typical clinical reasoning, some predictors will be used and some will be left out (see Figures 1 and 2). This would make some of the elements of any model from regression analysis superfluous. By contrast, a decision tree method generates a logical flow diagram that resembles a tree (17). This triangulated diagram, with repeated partitioning of the original data into smaller groups (nodes) on a yes or no basis, resembles clinical reasoning.

In order to develop a decision tree model, we sought a much larger dataset than previous studies, through the Virtual International Stroke Trial Archive (VISTA) (18). This archive contains data from available trials on ICH. Our aim was to use binary and trichotomy decision trees to predict mild, moderate, and severe disability outcomes at 3 months following ICH.

## Materials and Methods

Virtual International Stroke Trial Archive contains data from clinical trials including both ischemic stroke and ICH trials (18). Patients or their legal surrogates had signed informed consents for participation in clinical trials. The data are released in de-identified manner so that the trials and treatment allocations are not known. As such the name of the trials and the dates in which the patients were recruited are not provided here. We searched VISTA records for the patients with ICH. The following fields were used for extraction of imaging data: volume of ICH, intraventricular hemorrhage, midline (septum pellucidum) shift, location (basal ganglia, lobar and infratentorial hemorrhage); baseline clinical data: GCS, physiological variables (systolic blood pressure, blood sugar level), demographic data (age, gender), risk factors (hypertension, diabetes, antiplatelet drugs), and 3 months outcome data [modified Rankin scale (mRS)].

### Decision Tree Analyses

We used recursive partitioning (known as *rpart*, which is a free version of CART and available from R foundation, http://cran.r-project.org/web/packages/rpart/rpart.pdf) to perform binary decision tree analyses. The binary term here refers to splitting the data into two major outcomes of interest: such as mild versus moderate to severe disabilities. The method uses a splitting rule built around the notion of “purity.” A node in the tree is defined as pure when all the elements belong to one class. When there is impurity in the node, a split occurs to maximize reduction in “impurity.” In some cases, the split may be biased toward attributes that contain many different ordinal levels or scales (19). Thus, the selection of an attribute as the *root* node may vary according to the splitting rule and the scaling of the attribute (19).

By contrast, the term trichotomy decision tree analyses refer to trees where the data are partitioned into three major outcomes of interest: mild, moderate, and severe disabilities. In this paper, the term trichotomy is preferred in order to avoid confusion with ordinal regression analysis. This analysis is performed using *rpartScore* (20). In this analysis, attributes with greater than 10% missing data are not used for the predictive model (20).

### Binary Decision Tree Models

In model 1, the variables included GCS in addition to age, gender, ICH volume at baseline, glucose, and systolic blood pressure. This step was taken in an attempt to replicate part of the ICH score (4) (with the exception of location of ICH). In model 2, we used baseline NIHSS instead of GCS and keeping the other predictors to be the same. The NIHSS was chosen here because it has recently been assessed for use in ICH (21).

### Trichotomy Decision Tree Models

The mRS ranges from 0 (no disability) to 6 (death). The mRS of 1 equates to minimal disability, 2 to mild disability, 3 to moderate disability, 4 to moderately severe disability, and 5 to severe disability and bed ridden. We partition the mRS as followed good (mRS 0–2), moderate (mRS 3–4), and severe disability and death (mRS 5–6) to keep the ordinal structure of the scale (22). In this analysis, we did not consider it clinically useful to separate outcome using the full range of the mRS since it would create many partitions with poor reproducibility. It has been suggested that mRS = 4 should be part of severe disability. We have also explored this analysis with a different partition of the mRS: good (mRS 0–2), moderate (mRS 3), and severe disability and death (mRS 4–6).

In model 3, we combined models 1 and 2. As such, both variables such as baseline NIHSS and GCS were used together in conjunction with the other demographic and physiologic variables.

### Validation

For the purpose of validation, we randomly extracted two-thirds of the original data to train the models and used the remaining one-third for validation. The discriminating ability of the models was assessed using the area under the receiver operating characteristic curve (AUC) and interpreted using the guidelines set by Hosmer and Lemeshow (23). An AUC of 0.5 is classified as no better than by chance; 0.6–0.69 provides poor discrimination; 0.7–0.79 provides acceptable (fair) discrimination; 0.8–0.89 provides good (excellent) discrimination; and 0.9–1.0 provides outstanding discrimination (23). The trichotomy decision tree models were assessed using the area under the ordinal ROC curve function. The differences in the AUC between the training and validation ROC curves were compared using the *Z*-score.

## Results

### Patient Demographics

Using the search criteria described in Section “Materials and Methods,” we identified 1,371 patients. From these data, there were 957 patients with complete data on important covariates such as age, gender, ICH volume, baseline NIHSS, baseline GCS, and systolic blood pressure. The mean age was 65.9 ± 12.3 years (63.7% males). The baseline ICH volume was 22.6 ± 22.1 ml, and the median was 15.2 (IQR 7.7–30.5).

The mean GCS was 13.7 ± 1.8 and the median was 15 (IQR 13–15). The mean baseline NIHSS was 13.7 ± 5.8 and the median NIHSS was 14 (IQR 9–18).

The frequency of hypertension was 64.9% (data available on 842 of 957 patients) and diabetes was 13.9% (data available on 842 of 957 patients). The serum glucose was 8.7 ± 13.4 mmol/l (data available in 890 of 957 patients) and systolic blood pressure was 176.6 ± 30.1 mm Hg. Patients had CT scan early after onset with the average time to CT scan being 3.7 ± 1.2 h. Too few patients had data recorded on location of ICH (31%) or presence of intraventricular hemorrhage/IVH (37.7%) and midline shift (61%) to include these variables in our analyses.

### Relationship between NIHSS, GCS, and ICH Volume

There was a positive correlation between NIHSS at baseline and the ICH volume (Spearman rho 0.62, 95% bootstrap CI 0.57–0.67). There was negative correlation between NIHSS and GCS (spearman rho −0.60, 95% bootstrap CI −0.65 to −0.54), and between GCS and ICH volume (spearman rho −0.45, 95% bootstrap CI −0.510 to −0.39).

### Prediction of Mild Disability with Binary Decision Tree—Model 1 (Focusing on GCS, ICH Volume, and Demographic and Physiologic Variables)

The binary decision tree (Figure 1A) showed that lower ICH volume (<13.2 ml) followed by younger age (<66.5 years), serum glucose (≤9.0 mmol/l), and systolic blood pressure (<170 mm Hg) discriminated between mild disability (mRS 0–2) versus moderate-to-severe disability or death (mRS 3–6). The AUC for predicting mild disability was 0.79 (95% CI 0.73–0.85) in the training data and 0.66 (95% CI 0.57–0.75) in the validation data. The *Z*-score for comparison between the training and validation binary decision trees was statistically different, 2.27 (*p* = 0.02).

The binary decision tree (Figure 1B) showed higher baseline NIHSS (>12), older age (≥73.5), and blood sugar level (>6.4 mmol/l) discriminated between mild disability (mRS 0–2) versus moderate-to-severe disability or death (mRS 3–6). The AUC for predicting mild disability was in the training in validation data were 0.82 (95% CI 0.76–0.87) and 0.77 (95% CI 0.69–0.85). The *Z*-score for comparison between the training and validation binary decision trees was not statistically different, 0.94 (*p* = 0.3).

### Model 1 (Focusing on GCS, ICH Volume, and Demographic and Physiologic Variables)—Prediction of Disability with Trichotomy Decision Tree

The trichotomy decision tree (Figure 2A) showed that ICH volume, age, and serum glucose helped to separate the three classes of outcome. The AUC was 0.79 (95% CI 0.71–0.87) for the training data and 0.68 (95% CI 0.53–0.83) for the validation data.

The trichotomy decision tree (Figure 2B) showed that high baseline NIHSS (≥14) and age (≥69.5) defined the group with severe disability; if the age is below 69.5 years old, then it defines the group with moderate disability. The group with mild disability is defined as having lower baseline NIHSS (<8). Those patients with higher baseline NIHSS (≥8) and higher blood sugar level (≥6.5 mmol/l) have moderate disability. The AUC was 0.73 (95% CI 0.67–0.79) for the training data and 0.68 (95% CI 0.58–0.79) for the validation data.

### Prediction with Binary Decision Tree—Model 2 (Focusing on Baseline NIHSS, ICH Volume, and Demographic and Physiologic Variables)

The binary decision tree showed that NIHSS (<12.5), followed by age (<73.5 years old) and serum glucose (<6.35 mmol/l) discriminated between mild and moderate-to-severe disability. The AUC for predicting good outcome in the training group was 0.82 (95% CI 0.76–0.87). The AUC in the validation group was 0.77 (95% CI 0.69–0.85). The *Z*-score was 0.94 (*p* = 0.3), indicating no statistically significant difference between the training and validation binary decision trees.

### Prediction with Trichotomy Decision Tree—Model 2 (Focusing on Baseline NIHSS, ICH Volume, and Demographic and Physiologic Variables)

The trichotomy decision tree (Figure 2) showed NIHSS, age, and serum glucose helped to separate the three classes of outcome. The AUC for the training data was 0.73 (95% CI 0.067–0.79). The AUC in the validation group was 0.68 (95% CI 0.58–0.79). The *Z*-score was 0.76 (*p* = 0.4), indicating no statistically significant difference between the training and validation trichotomy decision trees.

### Model 3 (Baseline NIHSS, GCS, ICH Volume, and Demographic and Physiologic Variables)

This model performed the same as model 2 above.

We have also analyzed the data with a different partition of disability: mild (mRS 0–2), moderate (mRS 3), and severe (mRS 5–6). For the model with covariates such as NIHSS, ICH volume, and demographic and physiologic variables, the AUC for the training data was 0.61 (95% CI 0.53–0.69). For the model with covariates such as NIHSS, GCS, ICH volume, and demographic and physiologic variables, the AUC for the training data was 0.54 (95% CI 0.42–0.66).

### Prediction of Severe Disability and Death—Model 1 (Focusing on GCS, ICH Volume, and Demographic and Physiologic Variables)

**Figure 3:**
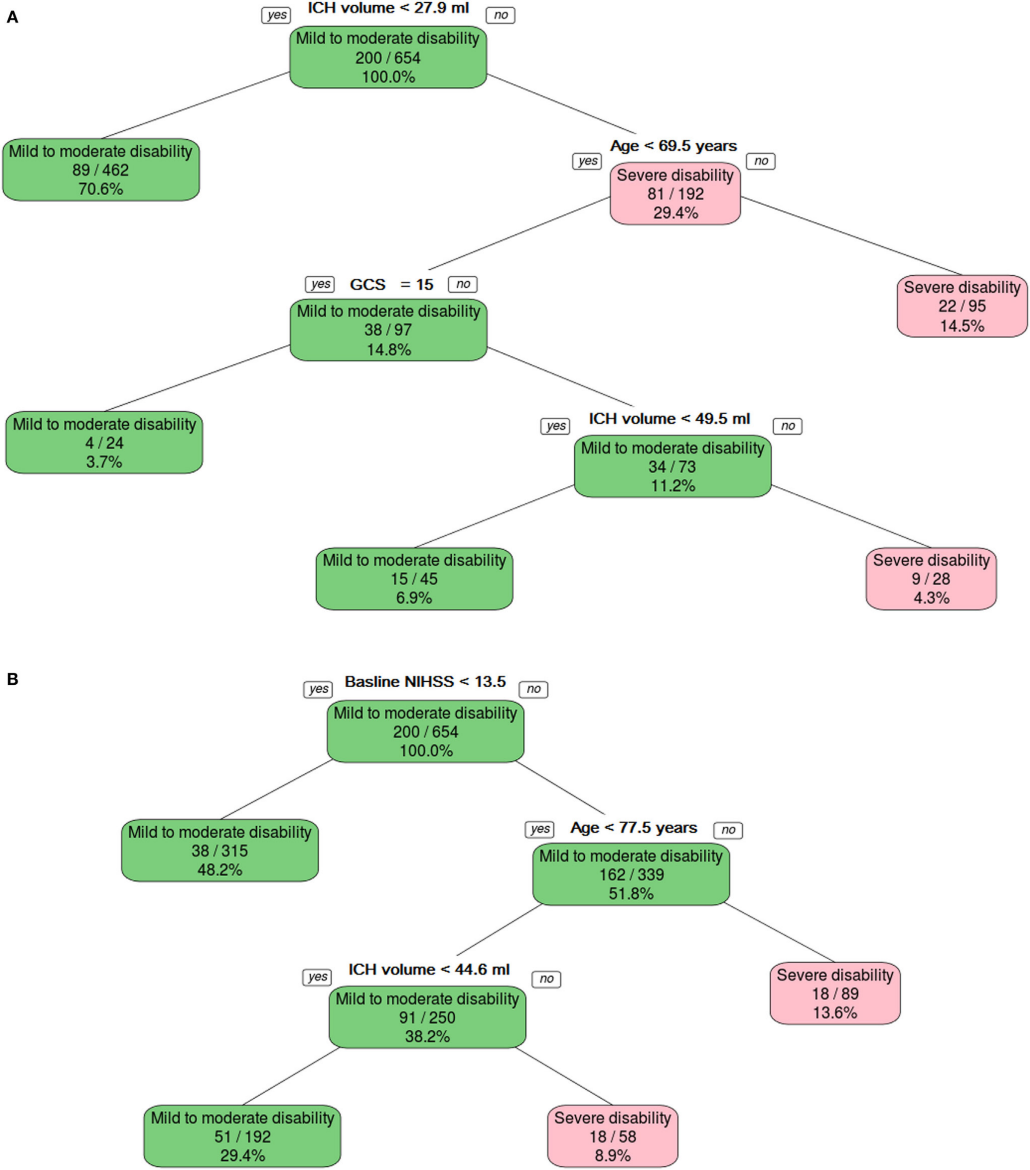
**The binary decision tree split the data into different categories on the basis of intracerebral hemorrhage (ICH) volume, age, and Glasgow Coma Scale (GCS)**. Panel **(A)** shows model developed without using NIHSS, and panel **(B)** shows model developed with NIHSS.

The binary decision tree showed that higher ICH volume (>27.9 ml) followed by older age (>69.5 years old) and low GCS (<15) discriminated between severe (mRS 5–6) and mild-to-moderate disability (mRS 0–4). The AUC was 0.80 (95% CI 0.75–0.86) in the training data and 0.79 (95% CI 0.70–0.88) in the validation data (Figure 3A). The *Z*-score for comparison was 0.17 (*p* = 0.9), indicating no difference.

### Combining NIHSS and GCS

The binary decision tree showed that higher NIHSS (≥13.5), followed by older age (≥77.5 years old) and larger ICH volume (44.6 ml) discriminated between severe disability and death versus mild-to-moderate disability (Figure 3B). The AUC was 0.84 (95% CI 0.79–0.89) in the training data and 0.78 (95% CI 0.69–0.87) in the validation data. The *Z*-score for comparison was 1.16 (*p* = 0.2), indicating no difference.

## Discussion

In this exploratory study, we have evaluated the use of trichotomy decision tree method for classifying outcome in ICH. These models were developed from clinical and imaging information that would be available at the time of patient presentation to the hospital. The trichotomy and binary methods are simple in both concept and usage, requiring very few attributes. These findings offer several methodological approaches to defining the group with moderate disability and who may benefit from participating in clinical trial.

### ICH Volume, GCS, and NIHSS

In this study, we had performed decision tree modeling with ICH volume and GCS rather than NIHSS; GCS had been used for developing prognostic model of mortality in patients with ICH (4, 5). Compared to NIHSS, GCS did not remain in the model for classifying mild disability probably because these patients would have GCS 14 or greater (see Figure 1) (15). By contrast, NIHSS provided a better partition for the data than both ICH volume and GCS for the prediction of mild disability (models 2 and 3). There were no differences in discrimination between models based on NIHSS and ICH volume and GCS in the models for severe disability.

### Comparison with Other Models

Our models have not relied on empirical threshold such as predefined ICH volume but are data driven (4, 5, 16). Previous models have empirically used ICH volume of 30 ml or greater to differentiate between severe disability and death (mRS 0–4) (2, 4, 5). In our study, an ICH volume approximately less than 30 ml was compatible with either mild or moderate disability, whereas an ICH volume less than 13 ml and glucose level less than 5.9 mmol/l occurred predominantly in patients with mild disability.

In this study, we were limited to data that have been entered into VISTA, and therefore, we do not have complete data on all variables that would be present at the time of patient’s presentation to hospital. Furthermore, we are limited in that the analysis in our project should not overlap with other VISTA projects. The aim here is to provide an exploration of the trichotomy decision tree method, and hence, the results of this analysis should be seen as an exploration and not a definitive predictive tool. The paucity of coded data on location of ICH and IVH extension meant that we cannot extend our analysis into the modifying effect of these variables on outcome in a way that has been done with ICH score (4). In the recent factor VIIa trial, there were 1.3% of patients with infratentorial ICH in the treatment arm and 2.6% in the placebo arm (13). It is possible that even if these data were available, there would not be enough of these cases to make inference about impact of ICH location. This low frequency may have been due to the requirement in such trial to exclude patients with plan for surgical evacuation within 24 h (13).

Similarly, data on other potential predictor of ICH growth, such as the spot sign, were not available for analysis (24). Our findings should be seen as example of what can be achieved with advancement in decision tree methods rather than finalized model for prediction. Consequently, we would seek to collaborate with other groups regarding application of such methods in the analysis of ICH outcome.

### Implications of ICH Volume in Clinical Trial Design

Our findings may have implication for clinical trial design, given they suggest that patients with ICH volume less than 13 ml are likely to have mild disability at 90 days. It is possible that results from the recent clinical trials may have occurred because they have included many cases of ICH with small volume (and which would have a good outcome regardless of treatment) (12, 13, 25). The recombinant factor VII trial had an average ICH volume 24 ± 26 and 23 ± 26 ml in the two treatment arms compared to 22 ± 24 ml in the placebo arm (13). In the recent trial of intensive blood pressure lowering in acute ICH, 39% of the treatment arm and 42% of the placebo arm had ICH volume less than 15 ml (12).

### Decision Trees

Decision trees have been used in machine learning tasks since the 1960s (17) but have been used sparingly in stroke (particularly ICH) for model development (15, 16). This methodology offers the advantage over standard regression methods in that it tolerates certain degree of missing number because the data are splitted using the available information for that attribute to calculate the Gini index (rather than the entire cohort). Despite this advantage, it is uncertain how much missing data the method would tolerate or whether it biases the results toward those cases that contain these data on these attributes. As such, we have conservatively chosen to avoid including attributes such as ICH location, IVH, and midline shift, where there are greater than 10% missing data. By contrast, attribute such as serum glucose has 7% missing data, but this number is with acceptable limit for the analyses. A potential disadvantage of the splitting rule used here is that it may be biased toward attributes that contain many different levels or scales. Thus, the selection of an attribute such as the *root* node (at the first split) may vary according to the splitting rule and the scaling of the attribute. Coincidentally in this case, the selection of ICH volume such as the *root* node corresponds to how we would design a clinical pathway and with previous information in the literature on the importance of ICH volume (2).

Decision tree methodology offers a second advantage over regression methods in that it resembles clinical reasoning and without the need to use equations or remembering the weights for each variable. The trade-off is that the user needs to remember the order of each variable in the partition. One may want to develop mnemonics to aid memorizing the order of the attributes. For the attributes in Figure 1A containing ICH volume, age, glucose, and systolic blood pressure, one may develop the mnemonics “I see Angels and Gods in the Sky” as a way to predict moderate and severe disability. In this study, the attributes are easy to remember as they are attributes that have been identified by other investigators to be important in ICH outcome (2, 4, 7–10). In certain situations, the outcome may be defined at the first, second, or third partitions of the tree. By contrast, models from regression methods attempt to use simultaneously all the attributes to arrive at prediction. Such redundancy in the attributes (as use in regression equation) can be seen in our decision trees where patients with very large ICH volume or high NIHSS are defined to have a poor outcome regardless of other variables.

### Trichotomy Decision Tree

The trichotomy decision tree method has not been used for clinical prediction rule in the stroke literature. The recent incorporation of this method in the R statistical package 2012 may be the reason why this has been the case (20). We were able to delineate the group who have moderate outcome by using the ability of trichotomy decision tree to split the data into three classes or more and without sacrificing accuracy AUC. A more cumbersome approach would be to perform sequential binary decision trees.

The success of the trichotomy decision tree points to a major advantage over regression method such as ordinal regression. That method uses a proportional odds model for analysis, which treats the odds of moving from one category to the other to be the same, i.e., the beta coefficient is constant among categories. That type of regression model applies the “parallel regression assumption” and may not be valid when the assumption of proportional odds does not hold true. In practice, this ideal situation is not always possible in stroke research and researchers have suggested alternative strategy such as the partial proportional odds model (with adjustment made for predictor variables which do not follow this assumption). Some investigators have suggested the combined use of ordinal regression and linear discriminant analysis to derive a prediction model (26, 27).

### Limitations

A limitation of this study is that it assumes mortality is due to disease and does not take into account variation in clinical practices such as do not resuscitate orders (28). The frequency of use of such orders impacts on mortality in ICH. Furthermore, the frequency of intensive blood pressure lowering in these trials was not known as the trials in this study were performed prior to the recent intensive blood pressure lowering trial (12). While this may be a limitation of this study, our finding that higher blood pressure is an attribute for prediction of poor outcome is in support of investigators evaluating the role of blood pressure lowering in ICH (12). Our study can be criticized for including subjects with mild stroke given that the median GCS was 13.7. However, the mean NIHSS of 13.7 suggests that these patients have moderately severe stroke deficit (defined as NIHSS 8–15) (29). Finally, there has been discussion on appropriate partition of the Rankin disability scale, particularly with regard to the threshold for moderate disability on the mRS. Based on recommendations to keep the ordinal structure of the mRS, we had chosen the partition as mild (mRS 0–2), moderate (mRS 3–4), and severe disability and death (mRS 5–6) (22). This partition severe disability and death as mRS 5–6 had been used in stroke trials (30, 31) and studies on prediction (32) and is consistent with recommendation by panel of experts (33). Nevertheless, we had also performed the analysis with the revised threshold for moderate disability as mRS equals to 3. Using this revised definition, severe disability and death correspond to mRS 4–6. However, the trichotomy decision tree analysis performed less well using the new threshold, suggesting our original selection for partitioning the mRS as appropriate.

## Conclusion

Decision tree methods can produce models with fair to excellent discrimination for disability outcome. We have shown several methodological approaches to identifying good and poor outcome groups. Both the binary and trichotomy methods provide equivalent discrimination of disability outcome after ICH. The trichotomy method can classify three categories at once, whereas this action was not possible with the binary method. These methods may be of use in clinical trial design and in re-evaluation of other trials for possible signals of efficacy (14).

## Author Contributions

Study concept and design: TP. Acquisition of data: TP and VISTA-ICH collaborators. Analysis and interpretation of data: TP, JC, and RB. Drafting of the manuscript: TP, VS, JC, RB, JL, BC, and HM. Critical revision of manuscript for intellectual content: all the authors. Statistical analysis: TP, JC, and RB.

## Conflict of Interest Statement

The authors declare that the research was conducted in the absence of any commercial or financial relationships that could be construed as a potential conflict of interest.

## VISTA-ICH Steering Committee

D. F. Hanley (Chair), K. Butcher, B. Gregson, S. Davis, K. R. Lees, P. Lyden, S. Mayer, K. Muir, and T. Steiner.
